# Cancer mortality inequalities in urban areas: a Bayesian small area analysis in Spanish cities

**DOI:** 10.1186/1476-072X-10-27

**Published:** 2011-04-15

**Authors:** Rosa Puigpinós-Riera, Marc Marí-Dell'Olmo, Mercè Gotsens, Carme Borrell, Gemma Serral, Carlos Ascaso, Montse Calvo, Antonio Daponte, Felicitas M Domínguez-Berjón, Santiago Esnaola, Ana Gandarillas, Gonzalo López-Abente, Carmen M Martos, Miguel A Martínez-Beneito, Agustín Montes-Martínez, Imanol Montoya, Andreu Nolasco, Isabel M Pasarín, Maica Rodríguez-Sanz, Marc Sáez, Pablo Sánchez-Villegas

**Affiliations:** 1 Servei de Sistemes d'Informació Sanitaria, Agència de Salut Pública de Barcelona, Barcelona, Spain; 2 CIBER Epidemiología y Salud Pública (CIBERESP), Parc de Recerca Biomédica de Barcelona, Barcelona, Spain; 3 Departament de Salut Pública, Facultat de Medicina, Universitat de Barcelona, Barcelona, Spain; 4 Universitat Pompeu Fabra, Barcelona, Spain; 5 Estudios e investigación Sanitaria, Departamento de Sanidad y Consumo. Gobierno Vasco, Vitoria-Gasteiz, Spain; 6 Observatorio de Salud y Medio Ambiente de Andalucía (OSMAN), Area de Salud Pública y Protección de la Salud, Escuela Andaluza de Salud Pública, Granada, Spain; 7 Servicio de Informes de Salud y Estudios, Instituto de Salud Pública, Dirección General de Salud Pública y Alimentación, Consejería de Sanidad, Comunidad de Madrid; 8 Servicio de Epidemiologia, Dirección General de Atención Primaria, Comunidad de Madrid, Spain; 9 Area de Epidemiología Ambiental y Cáncer, Centro Nacional de Epidemiología, Madrid, Spain; 10 Area de Desigualdades en Salud, Centro Superior de Investigación en Salud Pública de Valencia, Spain; 11 Instituto Aragonés de Ciencias de la Salud, Aragón, Spain; 12 Departamento de Medicina Preventiva e Saude Pública, Universidade de Santiago de Compostela, Spain; 13 Unitat d'Investigació en Anàlisi de la Mortalitat i Estadística Sanitaria, Departament d'Infermeria Comunitària, Medecina Preventiva i Salut Pública i Història de la Ciencia, Universitat d'Alacant, Spain; 14 Research Group on Statistics, Applied Economics and Health (GRECS), University of Girona, Spain

## 

**Figure 1:**
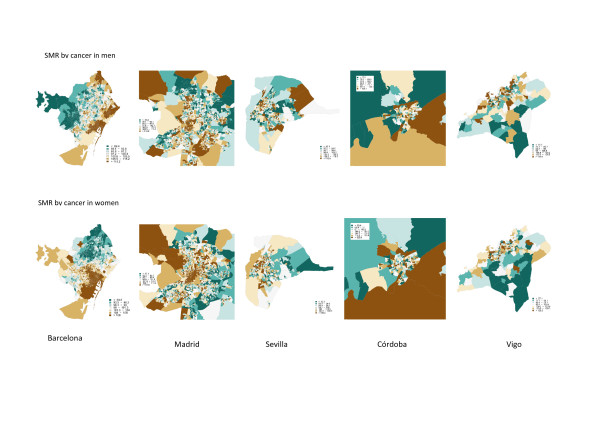
**(Figure two in original manuscript)**: Cancer mortality (smoothed Standardized Mortality Ratios) by census tract in men (top) and women (bottom) in Barcelona, Madrid, Sevilla, Córdoba and Vigo.

After publication of this work [1] it was brought to our attention that the map of Barcelona in Figure two (figure 1 here) was reversed. The final correct Figure is presented here.

We regret any inconvenience that this inaccuracy may have caused.
